# Enhancement of Forskolin Production Using Aeroponic Cultivation of *Coleus forskohlii* and the Impact on the Plant Phytochemistry

**DOI:** 10.3390/molecules29174215

**Published:** 2024-09-05

**Authors:** Audrey Le Cabec, Pierre-Eric Campos, Olivier Yzebe, Ronan Pelé, Cyril Colas, Emilie Destandau

**Affiliations:** 1 Institut de Chimie Organique et Analytique (ICOA), Université d’Orléans, CNRSUMR 7311, 45067 Orleans, France; audrey.le-cabec@univ-orleans.fr (A.L.C.); pierre-eric.campos@univ-orleans.fr (P.-E.C.); cyril.colas@univ-orleans.fr (C.C.); 2 Comité de Développement Horticole Région Centre (CDHRC), 45590 St-Cyr-en-Val, France; olivier.yzebe@cdhrc.fr (O.Y.); ronan.pele@cdhrc.fr (R.P.)

**Keywords:** aeroponic cultivation, mass spectrometry, metabolomics, molecular network

## Abstract

Accessing plant resources to extract compounds of interest can sometimes be challenging. To facilitate access and limit the environmental impact, innovative cultivation strategies can be developed. Forskolin is a molecule of high interest, mainly found in the roots of *Coleus forskohlii*. The aim of this study was to develop aeroponic cultivation methods to provide a local source of *Coleus forskohlii* and to study the impact of abiotic stress on forskolin and bioactive metabolite production. Three cultivation itineraries (LED lighting, biostimulant, and hydric stress) along with a control itinerary were established. The forskolin content in the plant roots was quantified using HPLC-ELSD, and the results showed that LED treatment proved to be the most promising, increasing root biomass and the total forskolin content recovered at the end of the cultivation period threefold (710.1 ± 21.3 mg vs. 229.9 ± 17.7 mg). Statistical analysis comparing the LED itinerary to the control itinerary identified stress-affected metabolites, showing that LEDs positively influence mainly the concentration of phenolic compounds in the roots and diterpenes in the aerial parts of *Coleus forskohlii.* Moreover, to better define the phytochemical composition of *Coleus forskohlii* cultivated in France using aeroponic cultivation, an untargeted metabolomic analysis was conducted using UHPLC-HRMS/MS analysis and molecular networks on both the root and aerial parts. This study demonstrates that aeroponic cultivation, especially with the application of an LED treatment, could be a very promising alternative for a local source of *Coleus forskohlii* leading to easy access to the roots and aerial parts rich in forskolin and other bioactive compounds.

## 1. Introduction

*Coleus forskohlii* (*syn. C. barbatus* var. *barbatus*) is an aromatic perennial plant belonging to the Lamiaceae family, native to tropical and subtropical regions including Brazil, East Africa, India, Myanmar, Nepal, Pakistan, and Sri Lanka [1,2]. In traditional Hindu and Ayurvedic medicine, *C. forskohlii* has been used to treat a variety of conditions such as heart disease, abdominal colic, respiratory disorders, insomnia, and convulsions [3].

The taxonomic classification of *C. forskohlii* has been ambiguous for decades and was only updated in 2019 [4] when the genus *Plectranthus* was divided into three distinct genera: *Plectranthus* sensu stricto, *Coleus*, and *Equilabium*, a decision further supported by the diversity of diterpenoids within these groups [5]. Studies between 1962 and 2019, which included the species *Plectranthus barbatus* as a synonym for *C. forskohlii*, should be reconsidered in light of this update [4].

Forskolin, discovered in the year 1974, is the main labdane diterpenoid identified in *C. forskohlii.* Its precursor, (13R)-manoyl oxide, was localized in the root cork, which explains why forskolin is mainly localized there [6,7]. Some studies, however, also mention the presence of forskolin in the aerial parts [8]. Forskolin is mainly known for its role as an activator of hormone-sensitive adenylate cyclase in intact cells or tissues, making it valuable for research on understanding cyclic AMP (adenosine monophosphate)-dependent physiological processes [6]. Forskolin is also used as a dietary supplement for fat loss as it increases cyclic AMP, enhances the use of body fat, and stimulates the basal metabolism and has been shown to be a lipolytic agent in topical application [9,10]. A large number of studies have investigated forskolin’s bioactivities and demonstrated anti-inflammatory properties in vitro and in vivo [11]. Forskolin could also be used as an anti-inflammatory agent to treat inflammation associated with obesity by inhibiting the expression of NFκB (nuclear factor-kappa B)-dependent genes [12].

Further studies on the root phytochemistry were carried out, and various diterpenoids, mainly forskolin derivatives, were identified [13]. One study compared the phytochemical profile of the genera *Coleus* and *Plectranthus* and revealed the predominant presence in the *Coleus* species of abietanes oxygenated at C-14, with formic acid or acetic acid as acyl groups, while in *Plectranthus*, isovaleric, senecioic, or hydroxybenzoic acid groups can also be found [5]. Other studies, focusing on the aerial parts of *P. barbatus* prior to the division of the genus, identified various diterpenoids [14,15] and some phenolic compounds with antioxidant properties [16]. However, characterization of the phytochemical profile of the aerial parts is very limited.

As forskolin and its derivatives are molecules of pharmaceutical, nutraceutical (sports nutrition, weight management supplement), and cosmetic (lipolytic agent) interest, the demand for *Coleus forskohlii* on the health market has increased, further enhanced by a growing consumer preference for natural products [10,17]. Therefore, the search for efficient methods for producing high-quality extract, rich in bioactive compounds, or powder of *Coleus forskohlii* is of great interest [18]. To meet growing market demand, the development of off-ground cultivation methods that provide easy access to the roots without plant sacrifice, thus allowing several harvests from the same plant, has emerged. While forskolin can be produced from hairy roots or callus cell cultures, these production methods, which require biological engineering, are considerably removed from the intact plant and can result in different metabolomic profiles [19,20,21].

The aim of this study was to develop aeroponic cultivation of *C. forskohlii* to provide a local, easy, and sustainable sourcing of *C. forskohlii*. In aeroponic conditions, plants grow in the air, out of the soil, giving easy access to the roots, and a nutrient solution is sprayed directly onto the roots to promote biomass development [22,23]. Aeroponic cultivation reduces the consumption of water, fertilizers, pesticides, and herbicides compared to hydroponics or geoponics, but as there is no solid support culture to absorb the excess of nutrients, developing the appropriate system parameters for plant growth is a challenge [24].

To achieve this objective, the French Horticultural Development Committee of the Centre-Val de Loire region (Comité de Développement Horticole de la Région Centre-Val de Loire; CDHRC) carried out, for the first time, different cultivation itineraries, involving different stresses or elicitations to assess their impact on plant biomass and metabolite production. Three different aeroponic culture itineraries, artificial lighting (LEDs), biostimulant, and hydric stress, in addition to the control itinerary were evaluated in this way. First, to assess the impact on the production of forskolin, as it is the main valuable compound targeted in *C. forskohlii*, root extracts were analyzed using HPLC-ELSD, and the forskolin content was determined using a calibration curve. Then, to gain greater insight into the plant composition under these growing conditions and therefore to produce a controlled quality extract, the phytochemical profile of the roots and aerial parts of *C. forskohlii* cultivated aeroponically in Région Centre-Val de Loire was investigated. Many health products (e.g., dietary supplements, cosmetics) on the market today contain *C. forskohlii* root extracts; therefore, it is important to have a good knowledge of the chemical profile of the extract to produce roots rich in forskolin and other bioactive compounds with interesting biological activities. Furthermore, as the aerial parts have been less explored than roots and are produced with aeroponic cultivation, this study could direct future valorization studies to transform this by-product into a co-product. The phytochemical profile was established using a molecular network that associates, in the same cluster, molecules presenting the same fragmentation pathway and that should belong to the same molecular family. This mapping associated with a database is very helpful for compound identification and extract comparison.

Lastly, to better assess the impact of artificial lighting (LEDs), which proved to be the most promising mode of forskolin production, on both forskolin and the other compounds, the roots and aerial extracts obtained with these conditions were further investigated using UHPLC-HRMS analysis with untargeted metabolomic analysis and were compared to the control modality. Statistical data treatments of the UHPLC-HRMS analysis of each part of the plant of the two modalities were performed to highlight potential differences between the two phytochemical profiles and identify compounds potentially affected by the growing conditions.

## 2. Results and Discussion

The main objectives of this study were to implement aeroponic culture conditions facilitating root harvesting, to develop a local and efficient sourcing of *C. forskohlii* and its main metabolite of interest, namely, forskolin, which is present mainly in the roots. To better qualify and valorize the cultivated plant and highlight the impact of the use of abiotic stresses during cultivation on the production of plant metabolites, the phytochemistry of the root and aerial parts was investigated.

### 2.1. Forskolin Quantification 

**Figure 1:**
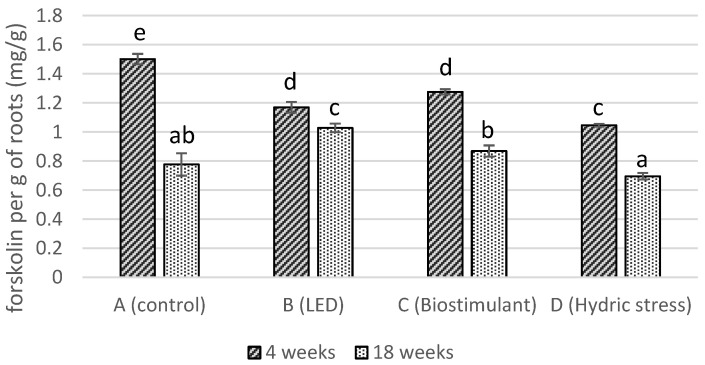
Comparison of the forskolin content per gram of *C. forskohlii* roots according to the time of cultivation and the modality (*n* = 3). Different letters indicate that means are significantly different at *p* < 0.05.

The forskolin content in roots from the four cultivation modalities was quantified and compared. The ethanolic extract of *C. forskohlii* was obtained by ultrasonic extraction and then dried to be solubilized at a known concentration of 5 mg/mL. A calibration curve (Figure S1) of forskolin was prepared between 25 and 400 µg/mL. The samples and a calibration curve were analyzed using HPLC-ELSD, and the forskolin content was calculated. Figure 1 presents the forskolin content per gram of *C. forskohlii* roots according to the time (4 and 18 weeks) and cultivation modality (A: control; B: LED; C: biostimulant; D: hydric stress). After 4 weeks of cultivation, the control showed the best result in terms of forskolin content with 1.50 ± 0.04 mg/g of dry roots, while the LED, biostimulant, and hydric stress did not have any positive impact on the concentration of forskolin. The amount of forskolin in cultivated *C. forskohlii* is comparable to that obtained in plants collected in natural habitats. For instance, the forskolin content of plants obtained from different locations in the Vidarbha region (Maharashtra, India) showed between 0.66 and 10.24 mg/g of forskolin in the dried plant [25]. After a few weeks of cultivation, the content of forskolin decreased in the roots, especially for the control modality, with a decrease of 49% in forskolin production. A seasonal impact on the content in labdane diterpenes was also recorded for *C. forskohlii* growth in an experimental field [26]. After 18 weeks, group A reached the same level as group D, which, after 4 weeks, was the one with the lowest percentage of forskolin. The use of a biostimulant (group C) slightly limited the drop in forskolin content (minus 32% after 18 weeks) and led to a higher content than group D after 18 weeks. The itinerary using LEDs was the most promising one, as forskolin production dropped by only 12%. The forskolin content of 1.03 ± 0.03 mg/g of dry roots after 18 weeks of cultivation under lighting compared to 0.78 ± 0.08 mg/g without suggests that the drop in forskolin is linked to the photoperiod.

**Figure 2:**
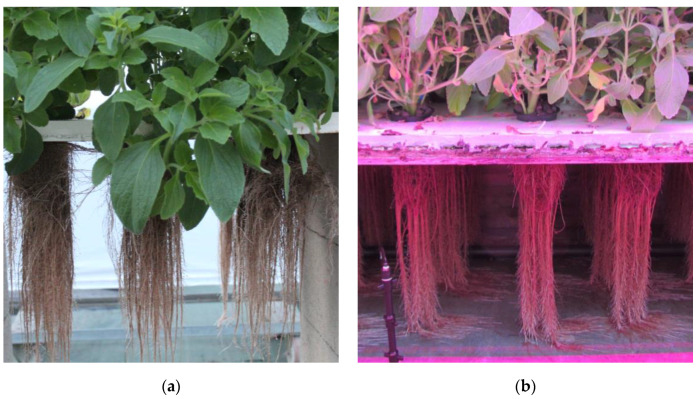
Aeroponic system at 18 weeks of cultivation with (**a**) itinerary A (control) and (**b**) itinerary B (LEDs).

After 18 weeks of cultivation, the total root biomass was collected, weighed, and the total quantity of forskolin produced was calculated. Table 1 compares the quantity of root biomass harvested and the total amount of forskolin recovered relative to the biomass produced. Neither hydric stress nor the biostimulant improved the quantity of biomass and forskolin compared to the control, while LEDs helped to multiply the biomass production by a factor of two, with 296.0 g of dry biomass obtained for itinerary A (control) and 690.8 g obtained for itinerary B (LEDs). By combining the amount of forskolin per gram of biomass (Figure 1) with the quantity of biomass produced, the itinerary using LEDs showed a significantly higher forskolin production with 710.1 mg of forskolin recovered, while the control condition led to the production of 229.9 mg, which triples the final quantity. Figure 2 shows plants of the two itineraries after 18 weeks of cultivation, demonstrating the better root development using LEDs (Figure 2b). The application of supplementary lighting with a photoperiod of 16 h increased the biomass production after a few weeks of cultivation. The biomass collected at week 4 in itinerary B was smaller than the one collected in itinerary A. This could be related to the natural photoperiod, which was longer in July when harvesting occurred after 4 weeks, compared to October when harvesting occurred after 18 weeks. Therefore, adding LEDs extended the natural photoperiod with a higher impact in October than in July and thus contributed to the increase in root biomass. Light influences root productivity, as shown with *Panax ginseng*, a plant from the Araliaceae family, where the beneficial effect of white LED lighting in a hydroponic cultivation system was demonstrated [27].

Itinerary B, which used LEDs, increased the total quantity of forskolin recovered after 18 weeks of cultivation, but forskolin may not be the only metabolite impacted by the stress. Therefore, an untargeted metabolomic study of the phytochemical profile was undertaken not only on roots but also on the aerial parts. As they are directly subjected to the stress, their composition can also be impacted. Furthermore, most previous studies focused on forskolin and therefore investigated only roots, but an investigation of both the roots and aerial parts could be valuable for the overall valorization of the plant.

### 2.2. Metabolomic Investigation

#### 2.2.1. Comparison of the Phytochemical Composition of Root and Aerial Parts

The aeroponic cultivation developed in this study under the control condition and LED lighting produced *Coleus forskohlii* with a forskolin content similar to that produced by *C. forskohlii* cultivated in the field in native countries.

As commercial products made from *C. forskohlii* are often root powders or extracts that contain many bioactive molecules and not only forskolin, it is important to determine the global phytochemical composition of the *C. forskohlii* produced in Région Centre-Val de Loire (France) using aeroponic cultivation. As *C. forskohlii* grew in soilless conditions without soil microorganisms and under different climate conditions, it is possible that the metabolomic composition was modified.

**Figure 3:**
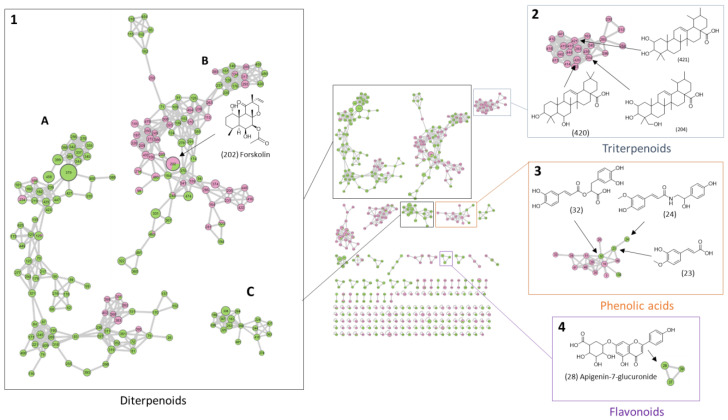
Molecular network of QC_R and QC_AP obtained from UHPLC-HRMS/MS data in positive ionization mode using GNPS with visualization performed using Cytoscape 3.10.2. The node size is proportional to the intensity of the ion. The color inside the nodes is a pie chart, with green for ions from aerial parts and pink for ions from roots. The structure of the annotated features characteristic of the compound family was added. Four main groups corresponding to four compound families were identified (1–4), group 1 contains three clusters named 1A, 1B and 1C.

Molecular networking is based on the principle that molecules with similar structures tend to have similar mass fragmentation patterns. These patterns can be represented as a graph where nodes represent ions and where edges are drawn between nodes if the similarity score between them exceeds a predefined threshold. Associated nodes define a cluster that should represent molecules belonging to the same molecular family. In molecular networking, several analyses can be joined and therefore compared to help in characterizing the phytochemical profile. The roots of *Coleus forskohlii* have been mainly studied due to their high concentration of forskolin [28,29,30]. As the aerial parts have been less studied, building a molecular network using both samples may make it possible to associate molecules of both samples in the same cluster, thus helping in the identification of the aerial parts. Moreover, comparing the composition of the root and aerial parts can reveal common molecules or compound families, facilitating the annotation; it can also highlight the phytochemical specificity of each part of the plant. Figure 3 shows the molecular network obtained on GNPS (global natural products social molecular networking) from the UHPLC-HRMS/MS data in positive ionization mode for QC_R (Quality Control Roots: a mix of root samples from itineraries A and B) and QC_AP (Quality Control Aerial parts: a mix of aerial part samples from itineraries A and B) to be able to detect the main compounds produced in the two conditions. Each color corresponds to a part of the plant, with green representing the aerial parts and pink representing the roots. Nodes are divided into two colors when the ion is present in both samples. The size of the pie chart corresponds to the ion proportion in each sample, while the size of the node represents the sum of the ion intensities in the two samples. In the center of Figure 3, a global overview of the network is shown, with 490 nodes, including the singletons. Three clusters (square 1 on the left part of Figure 3) are dedicated to diterpenoids, with one cluster (1B) including as many molecules from roots as molecules from aerial parts. This cluster includes forskolin (feature 202), which is also present in the aerial parts but in a smaller amount. Although this cluster contains ions from both roots and aerial parts, most of the compounds are found either in the roots or in the aerial parts specifically. This, along with the fact that the other two clusters (1A and 1C) of diterpenes mainly consist of compounds extracted from the aerial parts, indicates that the aerial parts are a source of a wider diversity of diterpenes, with different skeletons. Additionally, a cluster dedicated to triterpenoids was also highlighted for the roots (square 2 on the right of Figure 3). A few phenolic compounds (square 3 on the right of Figure 3), including rosmarinic acid (feature 32), were identified, along with a small cluster of flavonoids containing apigenin-glucuronide (feature 28) (square 4 on the right of Figure 3) in the aerial parts. Phenolic compounds contribute to the antioxidant properties of the plant. Rosmarinic acid and scutellarein 4′-methyl ether 7-O-glucuronide, both identified in the aerial parts of *P. barbatus*, have been shown to be correlated with the antioxidant activity of a water extract [16].

The overall composition of the root and aerial parts of plants grown in an aeroponic system is close to that described in plants grown in more conventional systems, with diterpenoids representing a large part of the composition of *C. forskohlii* and the presence of a few phenolic compounds. Based on this molecular network, a deeper exploration of the phytochemistry and annotation of the top 25 compounds impacted by the use of LEDs during cultivation (Section 2.2.2) was performed.

#### 2.2.2. Identification of Compounds Impacted by LEDs

In order to identify which metabolites were the most impacted by the addition of LEDs during cultivation, supervised multivariate analyses were employed. A PLS-DA (Partial Least Squares Discriminant Analysis) was performed using the peak list extracted from MetaboScape, and the top 25 Variables Important in Projection (VIP score > 1) were investigated.

**Figure 4:**
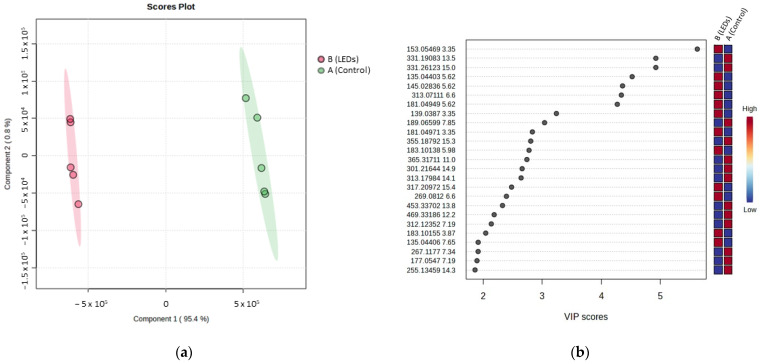

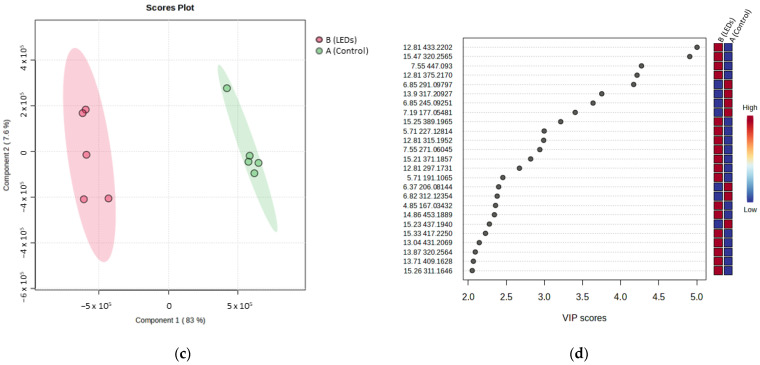
Supervised multivariate analysis based on untargeted UHPLC-HRMS analyses in positive ionization mode. (**a**) PLS-DA of itinerary A and B for root extracts and (**b**) the top 25 associated VIP scores; (**c**) PLS-DA of itinerary A and B for aerial part extracts and (**d**) the top 25 associated VIP scores.

Figure 4a presents the PLS-DA model based on the UHPLC-HRMS analyses in positive ionization mode of the root samples for control and LED conditions after 18 weeks of cultivation. Component 1 describes 95.4% of the variance, while component 2 represents 0.8%. The model achieved a quality prediction of R^2^ = 0.99 and a predictive ability of Q^2^ = 0.99. As shown in Figure 4a, two distinct groups can be easily distinguished in component 1, with itinerary A (control) on the right and itinerary B (LEDs) on the left. The samples of each group are separated along component 2 that describes only 0.8% of the variance, indicating the good similarity of samples belonging to the same modality. Figure 4b shows the top 25 VIPs associated with group separation on component 1, with 12 molecules being more intense in the LED cultivation itinerary and 13 more intense in the control condition.

In order to identify the VIPs, the molecular formula was first determined, and MS/MS data were employed to obtain fragmentation information. 

Table 2 and Table 3 summarize the compound information for the roots and aerial parts, respectively, including chromatographic information, the itinerary in which each compound is more present, the calculated molecular formula, and a putative annotation when possible. To increase the confidence level of the proposed identification, information from HRMS/MS was included; molecular formulae were searched in the LOTUS database from both the *Plectranthus* and *Coleus* genera, and HRMS/MS data were computed using SIRIUS 5.8. 

For root samples in Table 2, VIPs 1, 8, and 10 are all in-source fragments corresponding to compound **R1** with a t_R_ of 3.35 min and [M + H]^+^ at *m/z* 199.0598, suggesting a molecular formula of C_9_H_10_O_5_. The SIRIUS putative annotation suggested vanillylmandelic acid, a phenolic compound found in Lamiaceae [31]. The fragmentation spectrum shows ions at *m/z* 153.0543 and *m/z* 135.0434, corresponding to the losses of a carboxylic acidic function (46 u) and of a water molecule (18 u).

VIP 2 corresponding to compound **R12** at 13.5 min with a [M + H]^+^ ion at *m/z* 331.1908 suggests the molecular formula C_20_H_26_O_4_. An abietane diterpenoid derivative, the 14-hydroxytaxodione corresponding to this molecular formula was identified in *Plectranthus grandidentatus* [32]. 

VIP 3, which is a [M + Na]^+^ adduct, corresponds to compound **R17** at *m/z* 331.2612 with a t_R_ of 15.00 min and indicates a molecular formula of C_20_H_36_O_2_, consistent with a fatty acid derivative. In the molecular network, the corresponding node is a singleton, which does not give any further information on the structure of compound **R17**_._

VIPs 4, 5, and 7 are all associated with compound **R3** at a t_R_ of 5.62 min with a [M + H]^+^ at *m/z* 181.0494, which corresponds to the molecular formula C_9_H_8_O_4_. The fragmentation spectrum shows ions at *m/z* 163.0393 and 145.0282, suggesting the successive loss of H_2_O (18 u) due to hydroxyl functions, and *m/z* 135.0438 corresponding to the loss of a carboxylic acid function with another loss of the hydroxyl group with a fragment at *m/z* 117.0333. The molecular formula and fragmentation pattern suggest that this compound corresponds to caffeic acid, with a SIRIUS score of 100.00%. Caffeic acid is a phenolic compound commonly found in plants, including in the genus *Coleus* [33]. The node corresponding to **R3** is located in cluster 3 (Figure 3) dedicated to phenolic compounds.

VIPs 6 and 17 correspond to compound **R5** at a t_R_ of 6.60 min with the [M + H]^+^ at *m/z* 313.0711, indicating a molecular formula of C_17_H_12_O_6_. No compound in the genus *Plectranthus* or *Coleus* corresponds to this molecular formula, but SIRUS suggested that the compound could be 4-(3,4-Dihydroxyphenyl)-6,7-dihydroxynaphthalene-2-carboxylic acid with a score of 90.26%.

VIP 9 corresponds to **R9** with *m/z* 189.0659 and a t_R_ of 7.85 min. No information from the mass spectrum enabled the molecular ion to be identified; therefore, no molecular formula or annotation can be suggested. 

VIP 11 corresponds to compound **R18** with *m/z* 355.1879 and a t_R_ of 15.30 min. *m/z* 355.1879 had not been fragmented in MS/MS, which does not allow an annotation. Furthermore, no discriminating adduct that would have enabled the molecular formula to be calculated was found.

VIP 12 corresponds to **R4** at 5.98 min with a [M + H]^+^ at *m/z* 183.1015 and [M + Na]^+^ at *m/z* 205.0835, suggesting a molecular formula of C_10_H_14_O_3_. The molecular formula corresponds to a small phenolic compound, which is consistent with the retention time, but there is no further information available to suggest an annotation. 

VIP 13 corresponds to **R10** with a [M + H]^+^ *m/z* 365.3171 and a t_R_ of 11.00 min. The calculated molecular formula is C_22_H_40_N_2_O_2_, a nitrogen compound. 

VIP 14 corresponds to compound **R16** at *m/z* 301.2164 and at t_R_ of 14.90 min, and the corresponding adduct [M + Na]^+^ was identified with *m/z* 323.1981, which indicates a molecular formula of C_20_H_28_O_2_. In the literature, the two diterpenoids sugiol and barbatusol, corresponding to this molecular formula, were found in the genus Plectranthus [34,35].

VIP 15 corresponds to **R14** at a t_R_ of 14.10 min with a [M + H]^+^ at *m/z* 313.1798, suggesting the molecular formula to be C_20_H_24_O_3_. The corresponding node is located in a cluster of four nodes mainly from roots, suggesting a skeleton specific to this part of the plant. **R14** has a fragmentation pattern similar to that of diterpenoids and with a retention time consistent with other diterpenoid derivatives. 

VIP 16 corresponds to compound **R19** with *m/z* 317.2097 for [M + Na]^+^ and a t_R_ of 15.40 min, which suggests a molecular formula of C_18_H_30_O_3_. According to the retention time and the fragmentation pattern, **R19** may correspond to a fatty acid.

VIP 18 corresponds to compound **R13** with a t_R_ of 13.80 min and *m/z* 453.3370. The [M + H]^+^ and [M + NH_4_]^+^ at 489.3573 and 506.3840 suggested that 453.3370 *m/z* was [M − 2H_2_O + H]^+^. The molecular formula is C_30_H_48_O_5_, which corresponds to a triterpenoid; moreover, it is located in cluster 2, which is dedicated to triterpenoids. **R13** is annotated as tormentic acid previously identified in the genus *Coleus* [36] and in accordance with SIRIUS with a score of 87.45%.

VIPs 20 and 24 correspond to compound **R6** at a t_R_ of 7.19 min with the [M − H_2_O + H]^+^ at *m/z* 312.1235 and an in-source fragment at *m/z* 177.0547. The molecular formula of **R6** is C_18_H_19_NO_5_. SIRIUS suggested the compound to be N-feruloyloctopamine. Ion *m/z* 177.0540 corresponds to the ferulic derivative part of the molecule, while ion *m/z* 145.0279 is due to the loss of the methoxy group. Even with the nitrogen part, the node corresponding to **R6** node is located in cluster 3 dedicated to phenolic acids (ferulic derivative).

VIP 21 corresponds to **R2** at a t_R_ of 3.87 min with [M + H]^+^ at *m/z* 183.1015. The fragmentation pattern is similar to that of **R4** with the same molecular formula of C_10_H_14_O_3_. 

VIP 22 corresponds to compound **R8** at a t_R_ of 7.65 min with a fragment at *m/z* 135.0440 and a parent ion [M + H]^+^ at *m/z* 361.0922 and its corresponding adduct [M + NH_4_]^+^ at *m/z* 378.1188, which corresponds to a molecular formula of C_18_H_16_O_8_. This molecular formula corresponds to rosmarinic acid, with a node located in cluster 3 (phenolic acids), which is in accordance with the SIRIUS annotation with a score of 99.21%. The molecule has already been found many times in the Lamiaceae family including in the genera *Plectranthus* [37] and *Coleus* [16,38,39]. 

VIP 23 corresponds to **R7** with *m/z* 267.1177 and a t_R_ of 7.34 min. As no information from the mass spectrum made it possible to identify the molecular ion, no molecular formula or annotation can be suggested. 

VIP 25 corresponds to **R15** at 14.30 min, and *m/z* 255.1353 corresponds to a [M + Na]^+^ adduct with the corresponding [M + H]^+^ at *m/z* 233.1533, suggesting a molecular formula of C_15_H_20_O_2._ The retention time and molecular formula are consistent with a terpenoid derivative, but as the [M + H]^+^ was not fragmented, no further information was available to suggest an annotation.

Compounds with a retention time in the first part of the chromatogram seem to be more positively impacted by the use of LEDs and vice versa in the second half. LEDs seem to improve the intensity of phenolic compounds such as vanillylmandelic acid (**R1**), caffeic acid (**R3**), or rosmarinic acid (**R8**) in the roots of *C. forskohlii*, whereas they seem to decrease the amount of the most non-polar compounds such as diterpenoids and triterpenoids. As for *Ocimum basilicum*, from the same family as *C. forskohlii*, the artificial LED lighting seems to enhance the plant’s phenolic content, as phenolic compounds have UV radiation protection properties [40]. Forskolin, previously quantified in the roots (Section 2.1) and positively influenced by LED lighting, does not appear here in the top 25 VIPs. 

The same work was performed on the aerial parts of *C. forskohlii* to better understand the impact of LEDs on the whole plant as LEDs illuminate the aerial part. Figure 4a presents the PLS-DA model. Component 1 accounts for 83% of the variance and component 2 for 7.6%, with an R^2^ of 0.97 and a predictive ability Q^2^ of 0.98. In Figure 4c, two groups can be easily distinguished in component 1, with itinerary A (control) on the right and itinerary B (LEDs) on the left. Figure 4d presents the corresponding top 25 VIP scores, with 18 molecules being more intense for the cultivation itinerary using LEDs and 7 for the control itinerary.

VIPs 1, 4, 11, and 14 are related to **AP10** at a t_R_ of 12.81 min and correspond to [M + Na]^+^ at *m/z* 433.2202, [M − H_2_O + H]^+^ at *m/z* 375.2170, and two fragments, from the [M + H]^+^ at *m/z* 411.2377, *m/z* 315.1952 and *m/z* 297.1731. With a molecular formula of C_22_H_34_O_7_, **AP10** can be identified as forskolin. 

VIP 2 with *m/z* 320.2564 and a t_R_ of 15.47 min, corresponds to compound **AP20**. The calculated molecular formula is C_18_H_35_NO_2_. The fragmentation spectrum of the [M + H]^+^ at *m/z* 298.2753 of **AP20** indicates the loss of a molecule of water and the loss of the primary amide function with an ion fragment at *m/z* 245.2266 corresponding to fatty amide derivatives.

VIPs 3 and 12 correspond to **AP7** at a t_R_ of 7.55 min with a [M + H]^+^ at *m/z* 447.0930 and a molecular formula of C_21_H_18_O_11._ The ion at *m/z* 271.0604 corresponds to aglycone after the loss of the glucuronide moiety (176 u). **AP7** can be annotated as apigenin-glucuronide with a SIRIUS score at 96.74%, which is supported by the GNPS data library. The **AP7** node is located in cluster 4 with only three nodes, meaning that only two other similar flavonoid compounds present the same fragmentation pattern in the aerial parts of *C. forskohlii*. 

VIPs 5 and 7 correspond to compound **AP5** at a t_R_ of 6.85 min with a [M + H]^+^ at *m/z* 291.0979, which suggests that the molecular formula of **AP5** is C_14_H_13_N_2_O_4_. Fragment *m/z* 245.0921 corresponds to the loss of a carboxylic acid function (46 u), and fragment *m/z* 188.0702 corresponds to the loss of the N-malonyl part of the molecule, suggesting that compound **AP5** is N-malonyl-D-tryptophan with a SIRIUS score of 94.46%. 

VIP 6 corresponds to **AP14** with a [M + H]^+^ at *m/z* 317.2092 and a molecular formula of C_20_H_28_O_3_ associated with a diterpenoid. Several abietane diterpenoids correspond to this formula, such as 11,20-dihydroxysugiol identified in the aerial parts of *Plectranthus cyaneus*, royleanone identified in the whole plant of *Plectranthus grandidentatus*, and 20-Deoxocarnosol isolated from *Coleus barbatus* [41,42,43]. 

VIP 8 corresponds to compound **AP6** with a [M + H]^+^ at *m/z* 330.1339 and a t_R_ at 7.19 min. Compound **AP6** shows the same fragmentation pattern as **R6**, and both are located in cluster 3, with an ion source common fragment at *m/z* 177.0548. The molecular formula is C_18_H_19_NO_5_, and **AP6** is annotated as N-feruloyloctopamine, a hydroxycinnamic amide. 

VIPs 9 and 25 correspond to **AP18** with [M + H]^+^ at *m/z* 389.1965 and a fragment at *m/z* 311.1646 at a t_R_ of 15.25 min, which suggests a molecular formula of C_22_H_28_O_6_. The fragment at *m/z* 311.1646 indicates the loss of an acetate group together with the loss of a hydroxyl group (78 u). Seven different structures corresponding to abietane diterpenoids, especially abeoabietane diterpenoids or cycloabietane diterpenoids, were found in the genus *Plectranthus* using LOTUS [44,45,46,47,48]. The location of the corresponding node in cluster 1.A also supports this annotation. 

VIPs 10 and 15, which correspond to compound **AP2** at a t_R_ of 5.71 min, are two fragments at *m/z* 227.1281 and 191.1065 from the [M + NH_4_]^+^ adduct at *m/z* 406.2071. The molecular formula of compound **AP2** is C_18_H_28_O_9_. The fragment at *m/z* 227.1281 corresponds to the loss of a hexose moiety (162 u) with a molecular formula of C_12_H_18_O_4_. The aglycone is annotated as a hydroxyjasmonic acid, which implies that **AP2** is a hydroxyjasmonic hexose, a compound already described in the Lamiaceae family [49]. 

VIP 13 corresponding to **AP16** with a [M + H]^+^ at *m/z* 371.1857 and a t_R_ of 15.21 min has a molecular formula of C_22_H_26_O_5_. The node from the molecular network is located in cluster 1.A, which suggests that the compound should be annotated as a diterpenoid.

VIP 16, corresponding to compound **AP3**, is an ion source fragment at *m/z* 206.0814, itself originating from the [M + H]^+^ at *m/z* 252.0866 with a t_R_ of 6.37 min. The molecular formula of **AP3** is C_12_H_13_NO_5_. The node corresponding to **AP3** is located in the same cluster as **AP5**, suggesting a structural similarity between them. SIRIUS annotated **AP3** with a score of 86.64% as a dipeptide as 2-[(2-carboxyacetyl)amino]-3-phenylpropanoic.

VIP 17 corresponds to compound **AP4** with a t_R_ of 6.82 min and *m/z* 312.1235; it is a nitrogen compound with the molecular formula C_18_H_17_NO_4_. The fragment *m/z* 177.0547 corresponds to the ferulic derivative part of the molecule as described previously for **R6** and **AP6**. SIRIUS suggested that compound **AP4** is a hydroxycinnamic acid amide. 

VIP 18, which corresponds to compound **AP1** at a t_R_ of 4.85 min, is an in-source fragment ion from [M + H]^+^ at *m/z* 227.0551 with a molecular formula of C_10_H_10_O_6_. **AP1** corresponds to a phenolic acid with a putative annotation from SIRIUS at 64.38% of 4,6-dimethoxyisophthalic acid. 

VIP 19, corresponding to compound **AP15**, is a [M + Na]^+^ adduct with *m/z* 453.1889 and a t_R_ of 14.86 min. The calculated formula of C_24_H_30_O_7_ corresponds to a diterpenoid. A cycloabietane, (2R,2′S,3′R,4′*b*S,7′R,8′*aS*,9′S,10′S)-3′,10′-Diacetoxy-4′b,5′,6′,7′,8′,8′a,9′,10′-octahydro-9′-hydroxy-2,4′b,7′-trimethyl-8′-methylidenspiro[cyclopropan-1,2′(1′H)-phenanthren]-1′,4′(3′H)-dion, corresponding to this molecular formula was previously identified [46]. 

VIP 20, corresponding to **AP17** at *m/z* 437.1940, is a [M + Na]^+^ adduct of the [M + H]^+^ at *m/z* 415.2117 with a t_R_ of 15.23 min. The fragmentation pattern of the [M + H]^+^ corresponds to a diterpenoid, with a node located in cluster 1.A. The molecular formula of C_24_H_30_O_6_ corresponds to an acetylated derivative of a 6,7-secoabietane diterpene previously identified in the genus *Coleus* [50]. 

VIP 21 corresponds to the [M + Na]^+^ adduct of the compound **AP19** with a t_R_ of 15.33 min and *m/z* 417.2250. The calculated molecular formula of C_22_H_34_O_6_ is associated with different labdane diterpenes previously identified in *Plectranthus* and *Coleus*, including a derivative from forskolin, 9-deoxyforskolin [13], a labdane diterpenoid, which is consistent with the molecular network as the node of **AP19** is found in cluster 1.B (Figure 3) where forskolin is located. 

VIP 22 corresponds to **AP11** with a [M + H]^+^ at *m/z* 431.2069 corresponding to the molecular formula C_24_H_30_O_7_, identical to that of **AP15**, and a t_R_ of 13.04 min. The node is located in cluster 1.A associated with different diterpenes. As mentioned for **AP15**, a cycloabietane corresponding to this molecular formula was previously identified [46]. 

VIP 23 at a t_R_ of 13.87 min is a [M + Na]^+^ adduct with *m/z* 320.2564 corresponding to **AP13**. The calculated molecular formula is C_18_H_35_NO_2_. As for compound **AP20**, the fragmentation spectrum of the [M + H]^+^ at *m/z* 298.2753 indicates the loss of a hydroxyl function and of a primary amide function with an ion fragment at *m/z* 245.2266, which corresponds to a fatty amide derivative.

VIP 24 corresponds to the [M + Na]^+^ adduct of compound **AP12** with a [M + H]^+^ at *m/z* 387.1810 and a molecular formula of C_22_H_26_O_6_. The fragmentation of the [M + H]^+^ corresponds to a cycloabietane diterpenoid, coleon Z, as previously reported in *Coleus* spp. [51]. 

Itinerary A, with no LED lighting, contains more dipeptide and nitrogen compounds such as cinnamic acid amides, while itinerary B increases the content of almost all listed diterpenes, including forskolin (Compounds **AP10**, **AP11**, **AP12**, **AP16**, **AP17**, **AP18**, and **AP19**), which indicates that LEDs seem to stimulate the biosynthesis of diterpenoids in the aerial parts.

To summarize the results obtained by comparing the control and LED modalities on metabolite amount, phenolic compounds appear to be concentrated in the root and aerial parts when using LEDs. This may be explained by the fact that they are generally UV protectors, and a higher light stress could improve their production in order to defend the plant. We also note that nitrogen compounds are more intense in the itineraries without LED stimulation. A possible explanation is that due to a higher biomass production with LED stimulation, amino acids are totally consumed for the production of primary metabolites and are hence less available for the production of other nitrogenous derivatives. Concerning diterpenes, one of the main molecular families of *C. forskohlii*, their concentration is higher in the leaves under LED stimulation, with, in particular, an increase in the forskolin content. The effect of LEDs on the roots is less clear, however. Some of the diterpenes are more concentrated in the roots from itinerary B, while others are more concentrated in the roots from itinerary A. Furthermore, some families of compounds, such as triterpenoids, previously identified in the molecular network, appear to be unaffected.

## 3. Materials and Methods

### 3.1. Plant Cultivation

#### 3.1.1. Aeroponic System

The aeroponic cultivation of *C. forskohlii* presents several advantages such as a low consumption of water and easy access to the roots during plant growth. The aeroponic system used at the CDHRC consists of a 3 m long, 1.50 m wide cultivation tank. Polystyrene plates are placed on the tray to support the plants.

Cuttings are taken from mother plants grown at the CDHR in plastic pots under greenhouse conditions. Cuttings 5 to 8 cm high are made by cutting the base of the cutting above a node, starting from the stem apex of the aerial parts. Depending on plant development, between 1 and 3 cuttings can be made per stem. Care should be taken to ensure that leaves or leaflets are present at the top of the cutting. To prevent them from drying out, only 2 leaves are left on the cutting; the others are cut off with pruning shears. If the remaining leaves are too large, they are cut in half to prevent the cutting from dying.

The cuttings are then placed in the aeroponic system. Once the plants have roots of over 3 cm, they are placed in cultivation baskets filled with clay balls and then installed on the cultivation device. Each cultivation itinerary consists of 87 *C. forskohlii* plants, with a cultivation surface of 4.50 m^2^ for each itinerary, giving a cultivation density of 19.3 plants/m^2^. The system is housed in a glass greenhouse, which is kept frost-free, and a nutrient solution is diffused for 30 s every 5 min onto the roots using a pump system. A Coic-Lesaint mineral fertilization, based on a set conductivity of 1.5 mS/cm, is carried out. The system was set up on 7 June 2023 with a final collection on 17 October 2023.

#### 3.1.2. Cultivation Itinerary

To improve biomass production or the production of compounds of interest, different itineraries were set up: (A) A control itinerary in which no abiotic stress was applied. (B) An itinerary integrating complementary lighting with a photoperiod of 16 h of daylight. The lighting is LED (83% red, 17% blue), with an intensity of 150 µmol/m^2^/s. (C) An itinerary with weekly application of a foliar biostimulant (Laminaveg) at a dose of 2 L/ha. (D) An itinerary with daily application of water stress. The pump is turned off for a period determined by the wilting state of the plant.

#### 3.1.3. Harvesting and Drying

During harvesting, the roots of each plant were cut at 10 cm from their base and then dried in a greenhouse. The roots were harvested several times, after 4 weeks of cultivation and at the end after 18 weeks. The aerial parts of the plants were harvested (after 18 weeks) at the end of the cultivation and dried in an oven at 50 °C.

### 3.2. Phytochemicals

#### 3.2.1. Chemicals

The absolute ethanol (>99.7%), acetonitrile (HPLC gradient grade), and formic acid (99–100%) used for extraction and HPLC-ELSD were all purchased from VWR (Fontenay-sous-Bois, France) and were HPLC gradient grade. Ultrapure water was produced with a Milli-Q EQ7000 system equipped with an LC-Pak filter from Merck (Darmstadt, Germany). UHPLC-HRMS analyses were performed with water and LC-MS grade acetonitrile from Honeywell (Seelze, Germany). Forskolin (>99%) was purchased from Extrasynthese (Genay, France), and a stock solution at 1 mg/mL was prepared in methanol.

#### 3.2.2. Sample Preparation

An amount of 250 mg of powder of dried roots or aerial parts of *C. forskohlii* was extracted using ultrasound-assisted extraction with 12 mL of solvent during 1 h. The roots were extracted using 100% EtOH, while the aerial parts were extracted using a mixture of EtOH/water (80/20). The extract was then centrifuged during 10 min at 10,700× *g*, and the supernatant was filtered. The final extract was dried under nitrogen flow to obtain the dry crude extract. The dry extract was then dissolved with the same solvent used for extraction at a concentration of 5 mg/mL before further analysis.

All extractions for the quantification of forskolin in the roots were performed in triplicate (*n* = 3), while the extractions for the MS experiments, itineraries A and B of both the roots and aerial parts, were performed 5 times each (*n* = 5). For HRMS/MS, quality control samples were prepared: QC_AP, which is a mix of the supernatants from all aerial parts (itineraries A and B mixed), and QC_R, which is a mix of the supernatants from all roots (itineraries A and B mixed).

#### 3.2.3. HPLC-ELSD Quantification 

HPLC was performed with a Thermo Scientific Ultimate 3000 RSLC system using a binary pump, an automatic sampler, a thermostated column compartment, and an ELSD Sedex 100LT detector (Sedere, Olivet, France) in dynamic gain. The column was a Luna C18(2) (150 × 4.6 mm; 3 µm) (Phenomenex, Le Pecq, France). Gradient mode was used to separate the compounds. The mobile phase consisted of water (A) and acetonitrile (B), both acidified with 0.1% formic acid. The flow rate was fixed at 1 mL/min. Elution started with 10% B during 1.0 min. The amount of B increased up to 74% from 1 to 17.5 min and to 100% from 17.5 min to 18.5 min. The amount of B was maintained at 100% from 18.5 to 24.0 min and then decreased during 1.0 min to the initial conditions until 32 min.

A calibration range of forskolin was prepared in ethanol. Successive dilutions were made to obtain the 5 concentration solutions in the range 25–400 µg/mL (Figure S1).

All extractions for the quantification of forskolin in the roots were performed in triplicate (*n* = 3), and the data are presented as the mean ± standard deviation (SD). The statistical analysis was performed using XLSTAT (2017, Lumivero, Denver, CO, USA); differences between the samples were analyzed using one-way analysis of variance (ANOVA) and Tukey’s honestly significant difference with *p* ≤ 0.05.

#### 3.2.4. UHPLC-HRMS and UHPLC-HRMS/MS Analyses

Chromatographic analyses were performed with a Thermo Scientific Ultimate 3000 RSLC system using a binary pump, an automatic sampler, a thermostated column compartment, and a DAD detector (200–800 nm) (Dionex, Germering, Germany).

The column was a Luna Omega C18 (150 × 2.1 mm; 1.6 µm) (Phenomenex, Le Pecq, France). Gradient mode was used to separate the compounds. The mobile phase consisted of water (A) and acetonitrile (B), both acidified with 0.1% formic acid. The flow rate was fixed at 0.5 mL/min. Elution started with 5% B during 0.3 min. The amount of B increased up to 85% from 0.5 to 15 min and to 100% from 15 min to 16 min. From 16 to 21 min, the amount of B was maintained and then decreased during 0.2 min to the initial conditions until 23 min.

The MS and MS/MS experiments were carried out on a maXis UHR-Q-TOF mass spectrometer (Bruker, Bremen, Germany) using the data-dependent acquisition mode (DDA) with an electrospray ion source (ESI) working in positive ionization mode. Nitrogen was used as the drying gas at a flow rate of 9 L/min heated at 200 °C and as the nebulizing gas at a pressure of 2 bar. The mass spectra were recorded in the *m/z* range 50–1650 at 1.6 Hz for MS and MS/MS. The capillary voltage was set at 4.5 kV. Two precursor ions were selected per cycle, fragmented at two collision energies (15–35 eV), and averaged into one MS/MS spectrum. All extraction replicates were analyzed using MS for statistical analysis, while the QC sample of the aerial parts (QC_AP) and the QC sample of the roots (QC_R) were analyzed using MS/MS experiments for identification of metabolites (Figures S2 and S3).

### 3.3. Analyses of Treatments

#### 3.3.1. HRMS/MS Treatment

Analyses were converted to mzXML format using CompassXport (Bruker, Bremen, Germany). The mass spectrometry data were first processed with MZmine 3.8 [52]. Mass detections were fixed at 1.5 × 10^4^ for the MS1 and 8.0 × 10^2^ for the MS2 level. An ADAP chromatogram builder and resolver were employed, with a minimum of 4 consecutive scans at a threshold of 1.5 × 10^4^. Alignment was performed based on a *m/z* tolerance of 0.005 or 8 ppm and a retention time tolerance of 0.8 min. Isotope ^13^C grouper, gapfilling, and filtering were then performed. A feature list comprising 490 features between 2 and 16 min was finally obtained. A molecular network was then generated using GNPS [53] with the Feature-Based Molecular Networking (FBMN) workflow [54]. A molecular network was created in which the edges were filtered to have a cosine score above 0.7 and more than 6 matched peaks. The molecular network was then visualized using Cytoscape 3.10.2 software [55].

The molecular formulae were generated using elemental composition with a mass accuracy ≤ 5 ppm. The mzXML format of the analysis was computed using SIRIUS 5.8 [56] in order to suggest an annotation. Manual interpretation together with the use of databases such as LOTUS [57] and GNPS [53] helped to perform the annotation. 

#### 3.3.2. Statistical Treatment of HRMS Data

All the MS data were processed using DataAnalysis 4.4 (Bruker, Bremen, Germany). For MS experiments, all extractions of root and aerial parts were performed 5 times (*n* = 5) for a total of 20 samples. MetaboScape 4.0 software (Bruker, Bremen, Germany) was used to build the feature table, a matrix containing the retention times, *m/z*, and intensity for each ion corresponding to the following parameters: intensity threshold was fixed at 10,000, mass range was fixed from *m/z* 50 to 1650, and time range between 2.5 and 16 min. The matrix generated contained 1760 variables for root analysis and 1702 variables for aerial part analysis. Then, the MetaboAnalyst 6.0 platform was employed for statistical analysis. A reduced matrix was obtained using MetaboAnalyst 6.0 by conserving only the significant variables (*t*-test); this treatment allowed the obtainment of a reduced matrix with 371 variables and 441 variables for the roots and aerial parts, respectively. Then, multivariate analysis was performed on the reduced matrix using PLS-DA, and VIP scores were obtained to determine the most discriminant features. The EICs (extracted ion chromatograms) of each of the Top 25 VIPs are reported (Tables S1 and S2) and were further investigated using HRMS/MS data (with itineraries A and B of each part of the plant mixed for HRMS/MS analysis) in order to identify them. 

## 4. Conclusions

This study is the first time that the cultivation of *Coleus forskohlii* using an aeroponic system is reported, allowing a local sustainable production of forskolin. Furthermore, harvesting the roots is easier and does not harm the survival of the plant. The addition of LED lighting during the cultivation process, up to October towards the end of the cultivation period when the natural photoperiod decreases, stimulates the root biomass production of *C. forskohlii* and positively impacts the amount of forskolin recovered, resulting in a forskolin production that is three times higher than under control conditions (710.1 ± 21.3 mg vs. 229.9 ± 17.7 mg).

The global phytochemistry profile of both parts of the plant was evaluated using UHPLC-HRMS/MS data and molecular networking. The results highlighted a high diversity of diterpenoid compounds in both parts of the plant, with forskolin and its derivatives being common. In the aerial parts, a wider variety of diterpenoids was highlighted, represented by several dedicated clusters. Additionally, triterpenoids, phenolic compounds and nitrogenous compounds were present. The overall composition of the roots is similar to the one described in the literature, suggesting that extracts produced from plants using an aeroponic cultivation system will likely have similar bioactivity and, therefore, be valuable in the health market. For aerial parts, which have been less studied than roots, this study provides a first overview of the phytochemical composition. 

The aeroponic cultivation method with 16 h LED elicitation allows an increase in the production of *Coleus forskohlii* with an interesting yield of forskolin, making this method a very promising alternative for a local source of forskolin. Moreover, to identify the other metabolites affected by this stress, this study combined the statistical analysis of UHPLC-HRMS data (PLS-DA and VIP scores) with untargeted metabolomics using molecular networking and putative annotation obtained from GNPS, the LOTUS database, and SIRIUS computation of MS/MS data to support the annotation of the top 25 VIPs. These three databases, including experimental spectra, in silico fragmentation, and taxonomic information, improve the annotation of the molecules. The results showed that LEDs increase the phenolic compound composition in the roots, leading to higher amounts of rosmarinic acid or caffeic acid and the biosynthesis of diterpenes in the aerial parts such as forskolin, which are compounds of interest. 

A study examining the influence of LEDs with varying parameters (such as duration, photoperiod, and LED color) would be valuable for better understanding their impact on the biosynthetic pathways of forskolin and other bioactive compounds. This research would provide insights into these pathways and help to evaluate the effects of different lighting conditions, ultimately optimizing their use for improved compound production. As the aerial parts of the plant are also rich in diterpenes and phenolic compounds with this culture method, future research could focus on this part to explore this chemical diversity and include bioactivity assays in order to also valorize this by-product. 

## Figures and Tables

**Table 1:** Total biomass of *C. forskohlii* roots and forskolin production according to the cultivation itinerary after 18 weeks of cultivation. Percent decrease or increase in the value compared to itinerary A (control). For quantity of forskolin produced, data are presented as mean SD (*n* = 3); different letters indicate that means are significantly different at *p* < 0.05.

Cultivation Itinerary	A (Control)	B (LEDs)	C (Biostimulant)	D (Hydric Stress)
Dried root biomass (g) (Relative to the control)	296.0 -	690.8 (+133%)	264.0 (−11%)	305.2 (+3%)
Quantity of forskolin produced (mg) (Relative to the control)	229.9 ± 17.7 ^a^ -	710.1 ± 21.3 ^b^ (+209%)	229.4 ± 8.9 ^a^ (±0%)	212.4 ± 4.9 ^a^ (−8%)

**Table 2:** Identification of the top 25 VIP (VIP score > 1) metabolites discriminating root samples cultivated in control and LED conditions (ordered by t_R_).

Compound	t_R_	VIP Position	*m/z*	Adduct or Fragment	Itinerary	Calculated Molecular Formula [M]	Putative Annotation	Molecular Family
**R1**	3.35	1	153.0546	Fragments	B	C_9_H_10_O_5_	Vanillylmandelic acid	Phenolic acid
		8	139.0387					
		10	181.0497					
**R2**	3.87	21	183.1015	[M + H]^+^	B	C_10_H_14_O_3_	-	-
**R3**	5.62	7	181.0494	[M + H]^+^	B	C_9_H_8_O_4_	Caffeic acid	Phenolic acids
		5	145.0283	Fragments				
		4	135.0440					
**R4**	5.98	12	183.1014	[M + H]^+^	B	C_10_H_14_O_3_	-	-
**R5**	6.60	6	313.0711	[M + H]^+^	B	C_17_H_12_O_6_	4-(3,4-Dihydroxyphenyl)-6,7-dihydroxynaphthalene-2-carboxylic	Phenylpropanoids
		17	269.0812	Fragment				
**R6**	7.19	20	312.1235	[M − H_2_O + H]	A	C_18_H_19_NO_5_	N-Feruloyloctopamine	Cinnamic acid amides
		24	177.0547	Fragment				
**R7**	7.34	23	267.1177	-	A	-	-	-
**R8**	7.65	22	135.0440	Fragment	B	C_18_H_16_O_8_	Rosmarinic acid	Phenolic acids
**R9**	7.85	9	189.0659	-	A	-	-	-
**R10**	11.00	13	365.3171	[M + H]^+^	A	C_22_H_40_N_2_O_2_	-	-
**R11**	12.20	19	469.3319	[M + H]^+^	A	-	-	-
**R12**	13.50	2	331.1908	[M + H]^+^	A	C_20_H_26_O_4_	14-Hydroxytaxodione (abietane diterpene)	Diterpenes
**R13**	13.80	18	453.3370	[M − 2H_2_O + H]^+^	A	C_30_H_48_O_5_	Tormentic acid	Triterpenes
**R14**	14.10	15	313.1798	[M + H]^+^	A	C_20_H_24_O_3_	Abietane diterpene derivative	Diterpenes
**R15**	14.30	25	255.1353	[M + Na]^+^	A	C_15_H_20_O_2_	-	-
**R16**	14.90	14	301.2164	[M + H]^+^	A	C_20_H_28_O_2_	Sugiol / barbatusol	Diterpenes
**R17**	15.00	3	331.2612	[M + Na]^+^	A	C_20_H_36_O_2_	-	-
**R18**	15.30	11	355.1879	-	A	-	-	Fatty acid-
**R19**	15.40	16	317.2097	[M + Na]^+^	B	C_18_H_30_O_3_	Fatty acid derivative	Fatty acids

**Table 3:** Identification of top 25 VIP (VIP score > 1) metabolites discriminating aerial part samples cultivated following control and LED conditions (ordered by t_R_).

Compound	t_R_	VIP Position	*m/z*	Adduct or Fragment	Itinerary	Calculated Molecular Formula [M]	Putative Annotation	Molecular Family
**AP1**	4.85	18	167.0343	Fragment	B	C_10_H_10_O_6_	4,6-Dimethoxyisophtalic acid	Phenolic acid
**AP2**	5.71	10	227.1281	Fragments	B	C_18_H_28_O_9_	Hydroxyjasmonic acid hexose	Fatty acyl glycoside
		15	191.1065					
**AP3**	6.37	16	206.0814	Fragment	A	C_12_H_13_NO_5_	2-[(2-Carboxyacetyl)amino]-3-phenylpropanoic	Dipeptides
**AP4**	6.82	17	312.1235	[M + H]^+^	A	C_18_H_17_NO_4_	Hydroxycinnamic acid amide derivative	Cinnamic acid amides
**AP5**	6.85	5	291.0979	[M + H]^+^	A	C_14_H_13_N_2_O_4_	N-Malonyl-D-tryptophan	Dipeptides
		7	245.0925	Fragment				
**AP6**	7.19	8	177.0548	Fragment	A	C_18_H_19_NO_5_	N-Feruloyloctopamine	Cinnamic acid amides
**AP7**	7.55	3	447.093	[M + H]^+^	B	C_21_H_18_O_11_	Apigenin-glucuronide	Flavonoids
		12	271.0604	Fragment				
**AP10**	12.81	1	433.2202	[M + Na]^+^	B	C_22_H_34_O_7_	Forskolin	Diterpenes
		4	375.2170	[M − H_2_O + H]^+^				
		11	315.1952	Fragments				
		14	297.1731					
**AP11**	13.04	22	431.2069	[M + H]^+^	B	C_24_H_30_O_7_	Cycloabetiane derivative	Diterpenes
**AP12**	13.71	24	409.1628	[M + Na]^+^	B	C_22_H_26_O_6_	Coleon Z	Diterpenes
**AP13**	13.87	23	320.2564	[M + Na]^+^	B	C_18_H_35_NO_2_	N-Acyl amines derivatives	Fatty amides
**AP14**	13.9	6	317.2092	[M + H]^+^	A	C_20_H_28_O_3_	11,20-Dihydroxysugiol/ royleanone	Diterpenes
**AP15**	14.86	19	453.1889	[M + Na]^+^	B	C_24_H_30_O_7_	Cycloabetiane derivative	Diterpenes
**AP16**	15.21	13	371.1857	[M + H]^+^	B	C_22_H_26_O_5_	Abetiane diterpene derivative	Diterpenes
**AP17**	15.23	20	437.1940	[M + Na]^+^	A	C_24_H_30_O_6_	6,7-Secoabietane diterpene derivative	Diterpenes
**AP18**	15.25	9	389.1965	[M + H]^+^	B	C_22_H_28_O_6_	Abetiane diterpene derivative	Diterpenes
	15.26	25	311.1646	Fragment				
**AP19**	15.33	21	417.2250	[M + Na]^+^	B	C_22_H_34_O_6_	9-Deoxyforskolin	Diterpenes
**AP20**	15.47	2	320.2565	[M + Na]^+^	B	C_18_H_35_NO_2_	N-Acyl amines derivatives	Fatty amides

## Data Availability

The MS/MS and MS data presented in this study are openly available in Zenodo at https://doi.org/10.5281/zenodo.13685507. The molecular network obtained can be accessed at: https://gnps.ucsd.edu/ProteoSAFe/status.jsp?task=eb3ac66911f84c049379cd26715ef47a accessed on 4 September 2024.

## Supplementary Materials

The following supporting information can be downloaded at: https://www.mdpi.com/article/10.3390/molecules29174215/s1, Figure S1: Calibration curve for HPLC-ELSD quantification of forskolin: logarithm of peak area versus logarithm of concentration (µg/mL); Figure S2: Base peak chromatogram UHPLC-(+)ESI-MS/MS of QC_R (root extract); Figure S3: Base peak chromatogram UHPLC-(+)ESI-MS/MS of QC_AP (aerial part extract); Table S1: Extracted ion chromatogram of VIP compounds in roots (**R1**–**R19**); Table S2: Extracted ion chromatogram of compounds impacted in roots (**AP1**–**AP20**).
