# Supplementary material for: Enhancement of Forskolin Production Using Aeroponic Cultivation of Coleus forskohlii and the Impact on the Plant Phytochemistry
Source: Molecules. 2024 Sep 5;29(17):4215. doi: 10.3390/molecules29174215 (PMC11397331; doi:10.3390/molecules29174215)
Supplement: Supplementary file 1 [file molecules-29-04215-s001.zip › molecules-3172273-supplementary.pdf]

Figure S1 : Calibration curve of the forskolin quantification Log(A) = f(log(C)) with A the area and C the concentration in µg/mL

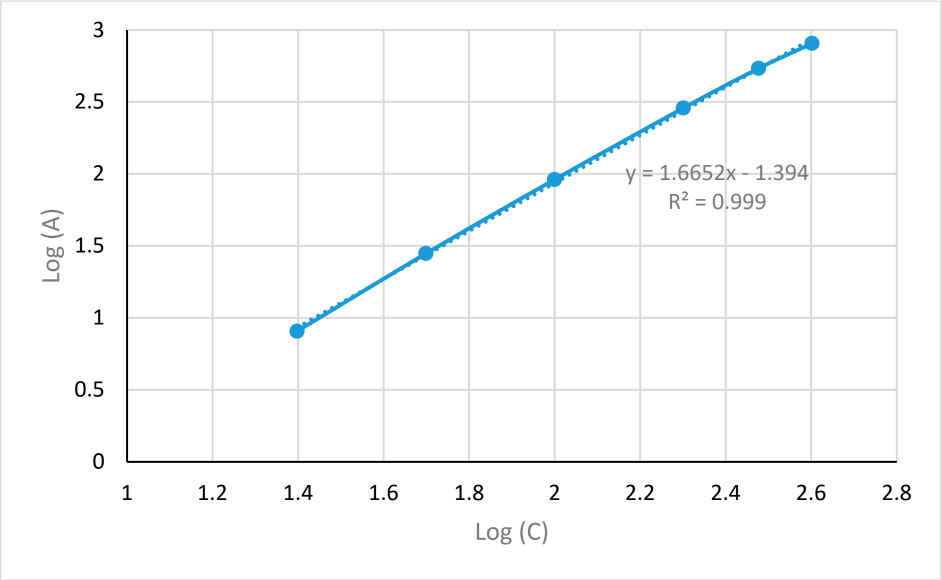

Figure S2: Base peak chromatogram UHPLC-(+)ESI-MS/MS of QC_R (roots extract)

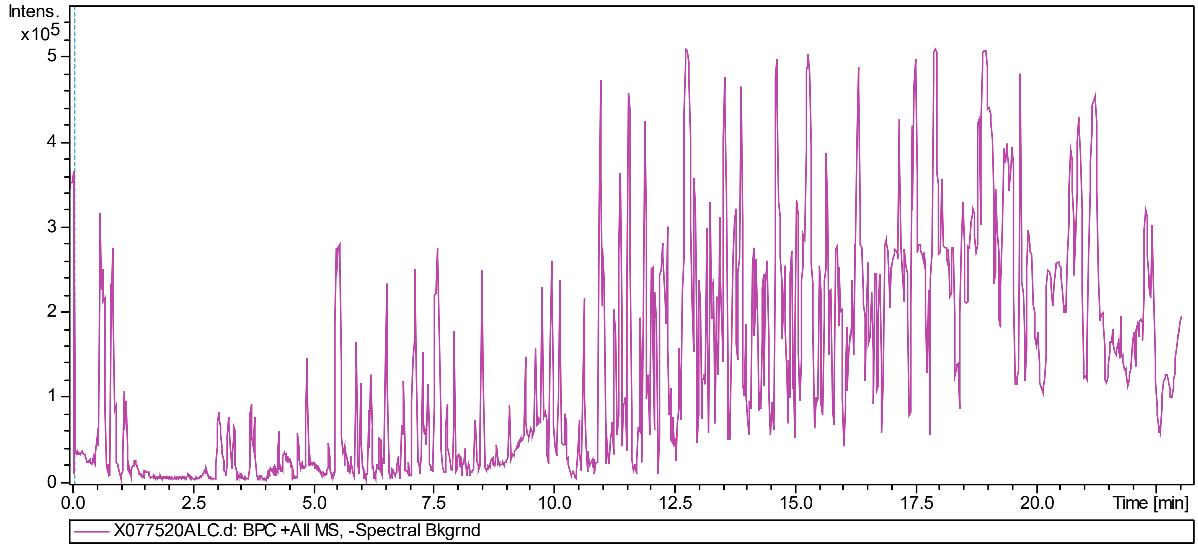

Figure S3: Base peak chromatogram UHPLC-(+)ESI-MS/MS of QC_AP (aerial part extract)

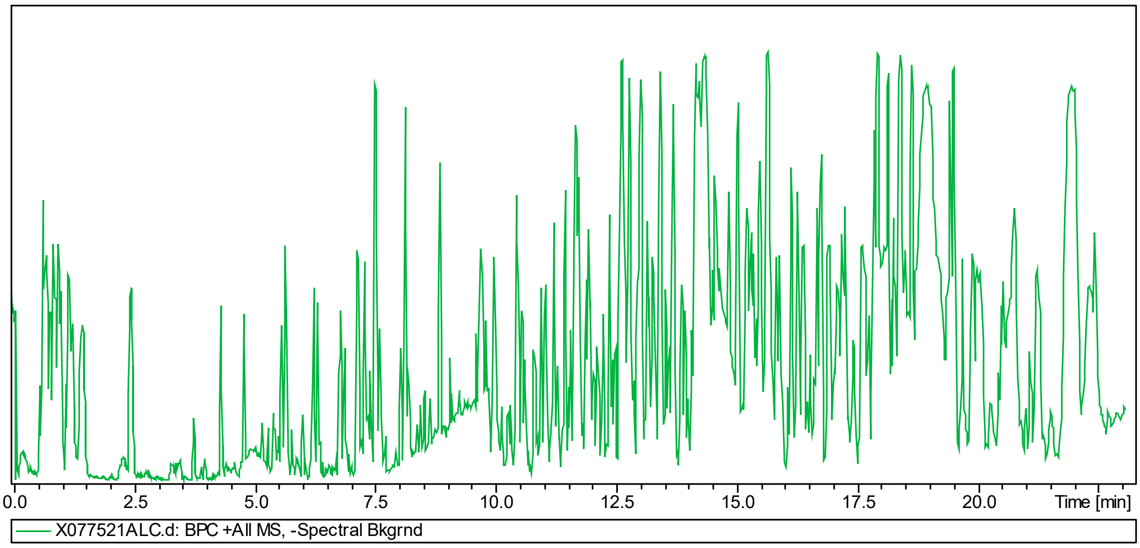

Table S1: Extract ion chromatogram of VIPs compounds in roots (R1-R19) (with yellow samples from itinerary B and blue sample from itinerary A)

Compound	Rt	VIP position	
R1	3.35	1	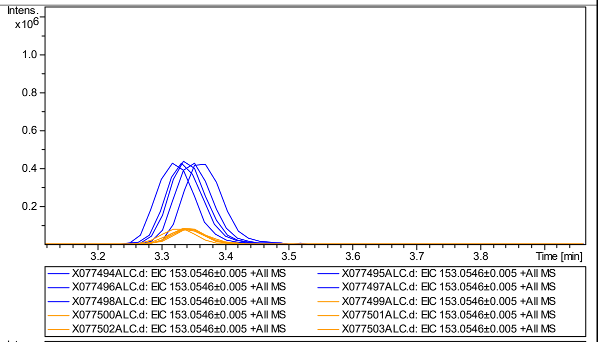
		8	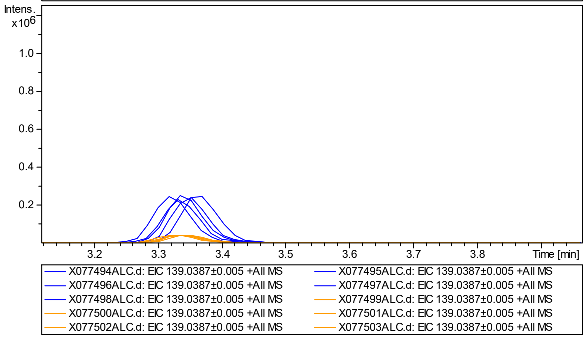

<table>
<tr><td></td><td></td><td>10</td><td>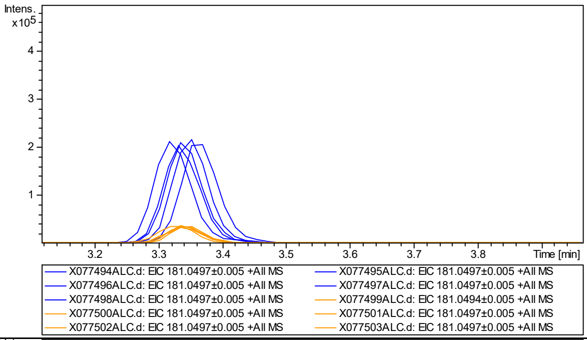
</td></tr>
<tr><td>R2</td><td>3.87</td><td>21</td><td>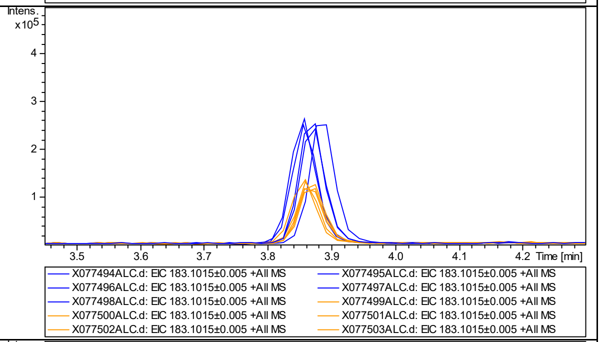
</td></tr>
<tr><td>R3</td><td>5.62</td><td>7
5
4</td><td>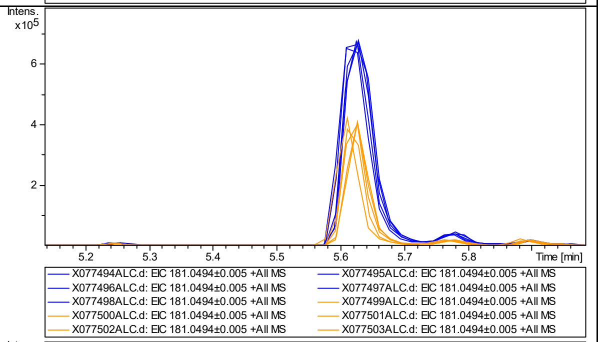

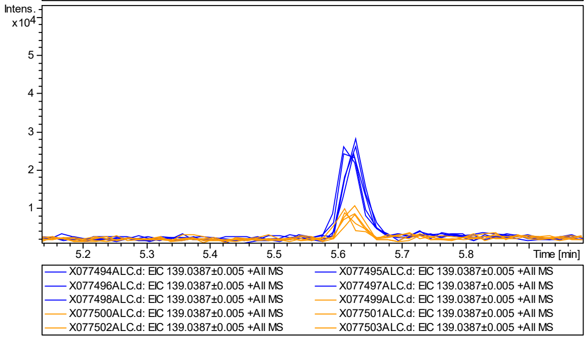
</td></tr>
<tr><td>R4</td><td>5.98</td><td>12</td><td>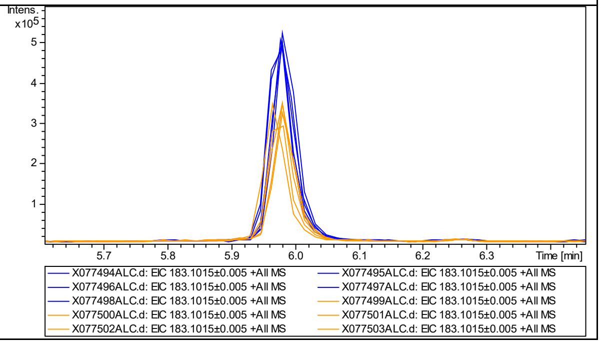
</td></tr>
</table>

<table>
<tr><td rowspan="2">R5</td><td rowspan="2">6.60</td><td>6</td><td>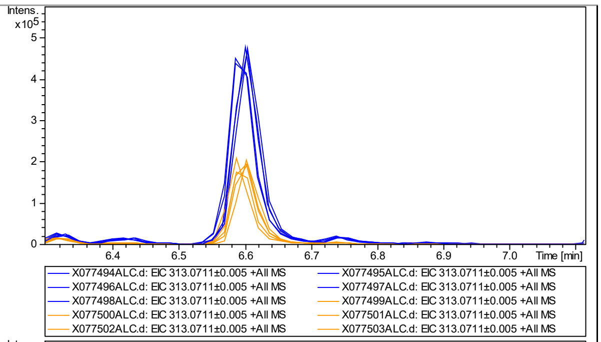
</td></tr>
<tr><td>17</td><td>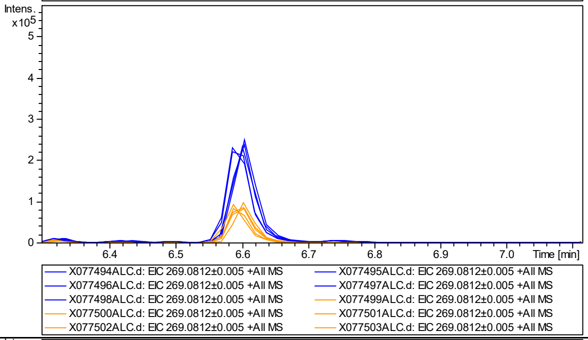
</td></tr>
<tr><td rowspan="2">R6</td><td rowspan="2">7.19</td><td>20</td><td>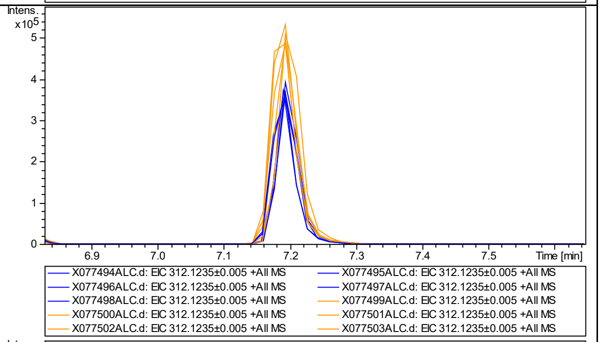
</td></tr>
<tr><td>24</td><td>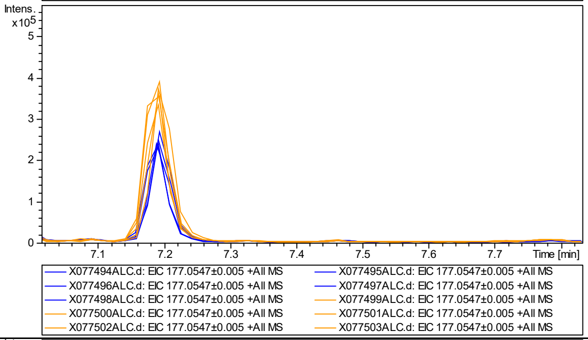
</td></tr>
<tr><td>R7</td><td>7.34</td><td>23</td><td>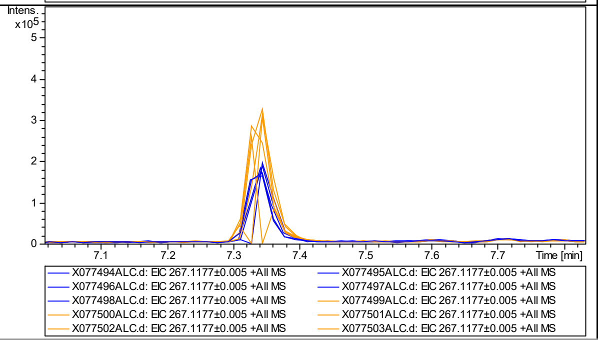
</td></tr>
</table>

**R8**	7.65	22	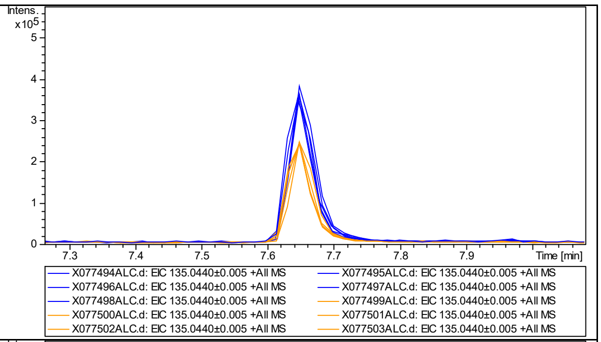
**R9**	7.85	9	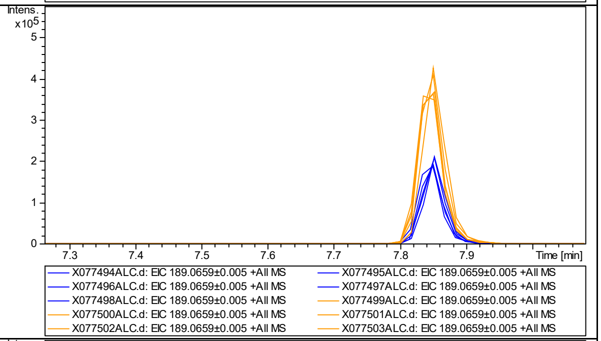
**R10**	11.00	13	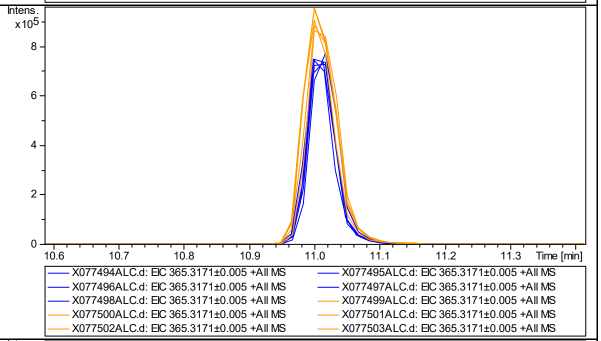
**R11**	12.20	19	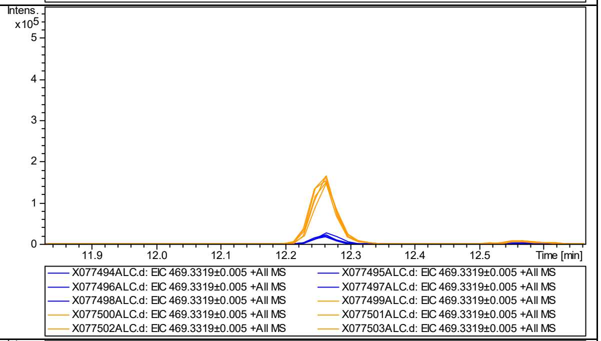
**R12**	13.5	2	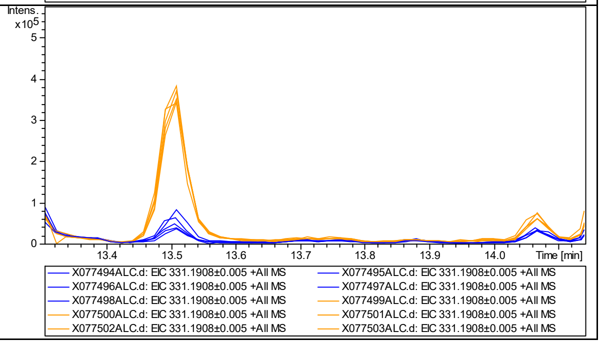

<table>
<tr><td>R13</td><td>13.80</td><td>18</td><td>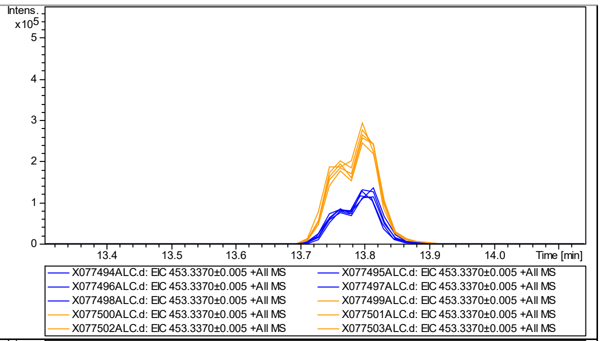
</td></tr>
<tr><td>R14</td><td>14.10</td><td>15</td><td>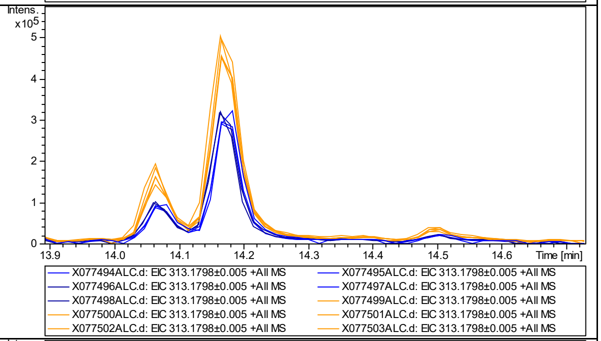
</td></tr>
<tr><td>R15</td><td>14.30</td><td>25</td><td>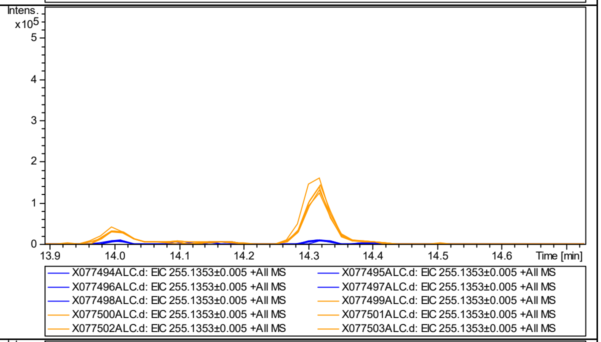
</td></tr>
<tr><td>R16</td><td>14.90</td><td>14</td><td>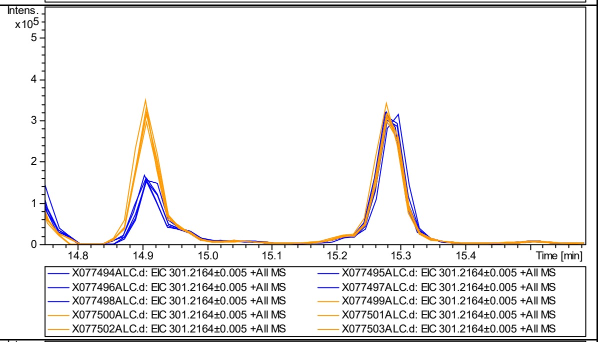
</td></tr>
<tr><td>R17</td><td>15.00</td><td>3</td><td>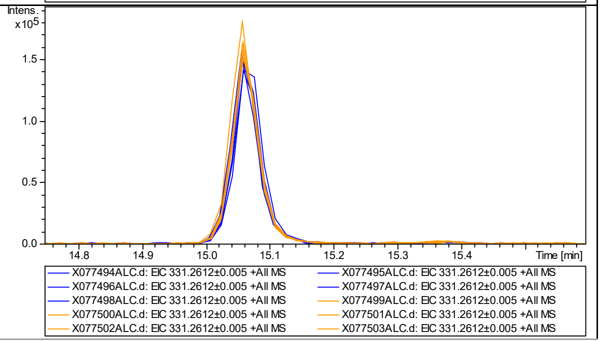
</td></tr>
</table>

**R18**	15.30	11	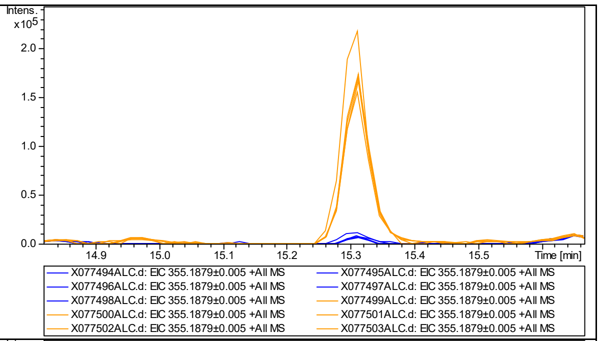
**R19**	15.40	16	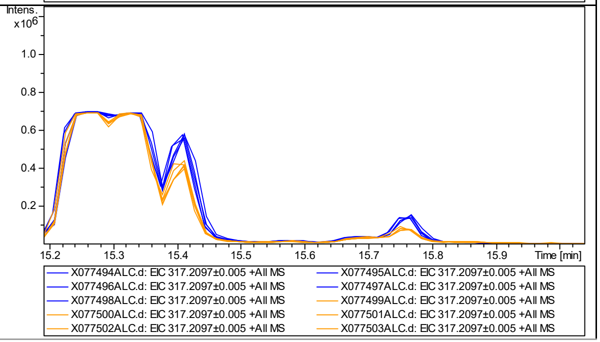

Table S2: Extract ion chromatogram of compound impacted in roots (AP1-AP20) (with yellow samples from itinerary B and blue sample from itinerary A)

Compound	Rt	VIP position	
**AP1**	4.85	18	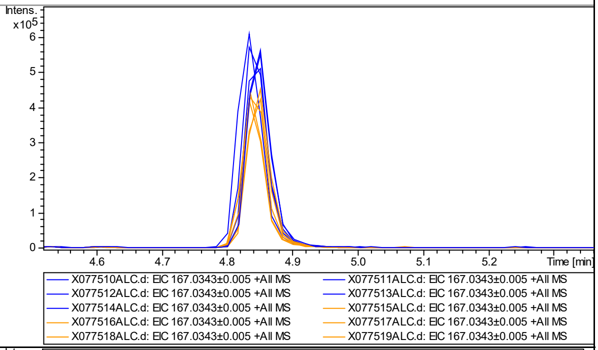
**AP2**	5.71	10	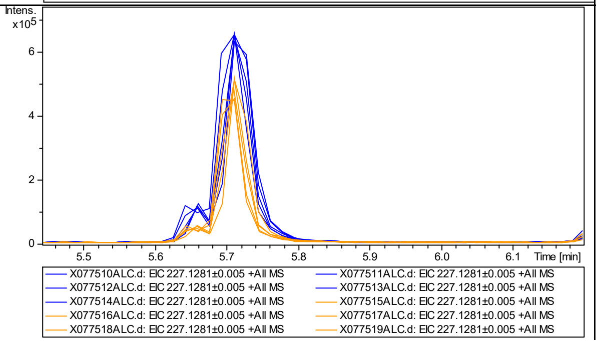

<table>
<tr><td></td><td></td><td>15</td><td>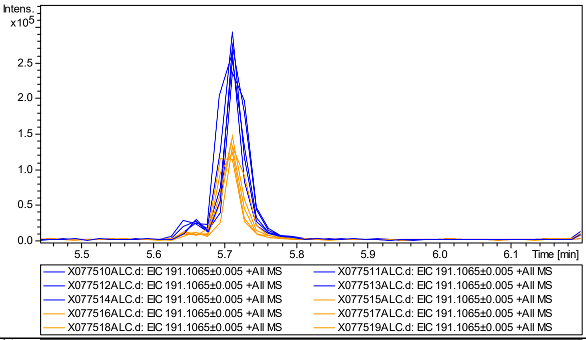
</td></tr>
<tr><td>AP3</td><td>6.37</td><td>16</td><td>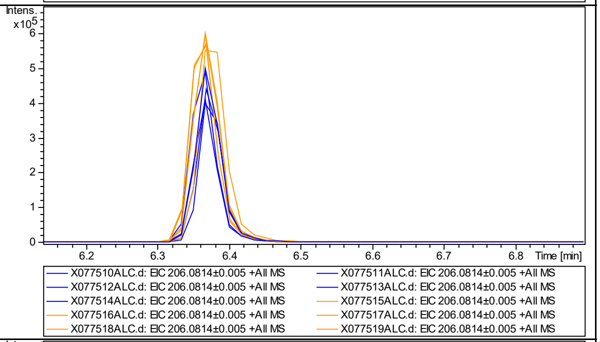
</td></tr>
<tr><td>AP4</td><td>6.82</td><td>17</td><td>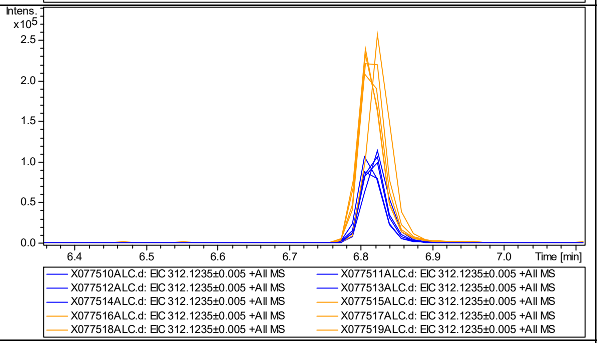
</td></tr>
<tr><td>AP5</td><td>6.85</td><td>5
7</td><td>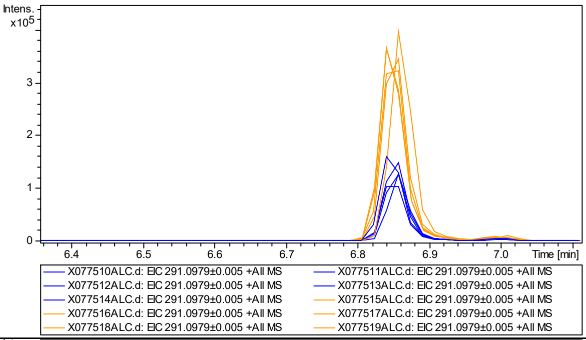
</td></tr>
<tr><td>AP6</td><td>7.19</td><td>8</td><td>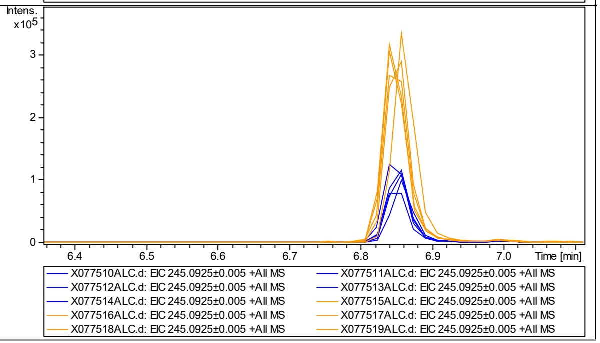
</td></tr>
</table>

**AP7**	7.55	3	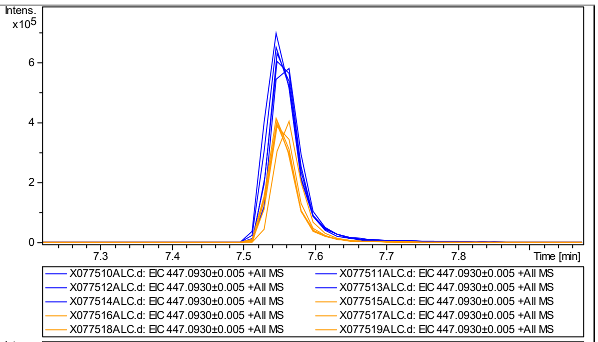
		12	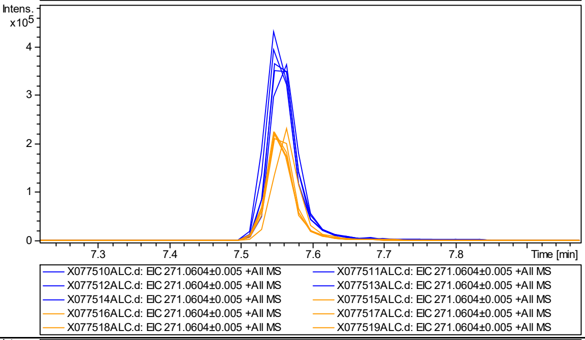
**AP10**	12.81	1	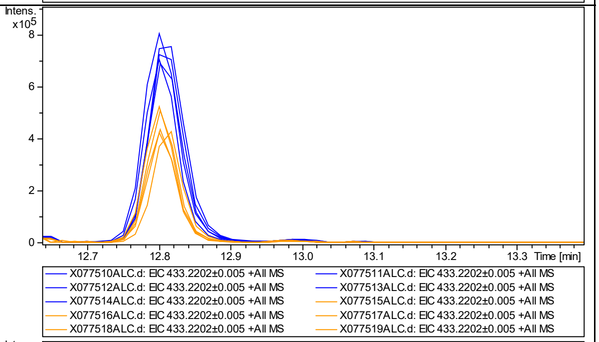
		4	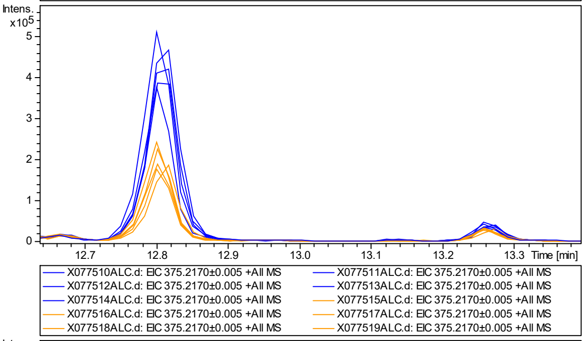
		11	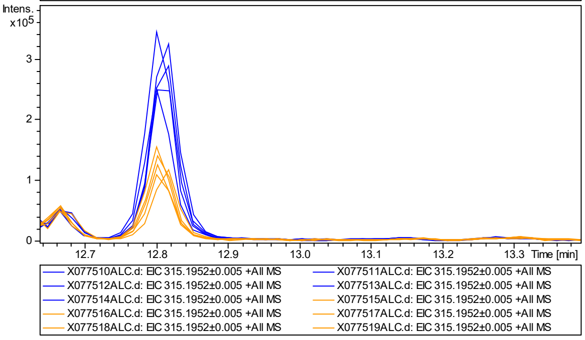

		14	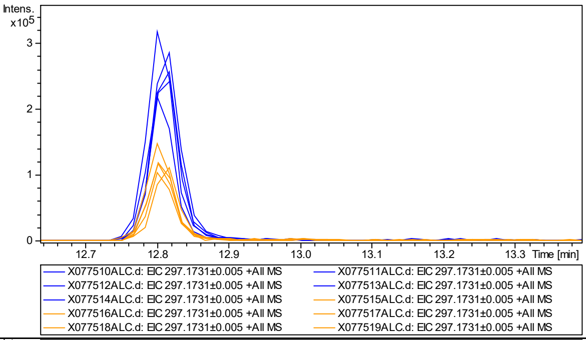
**AP11**	13.04	22	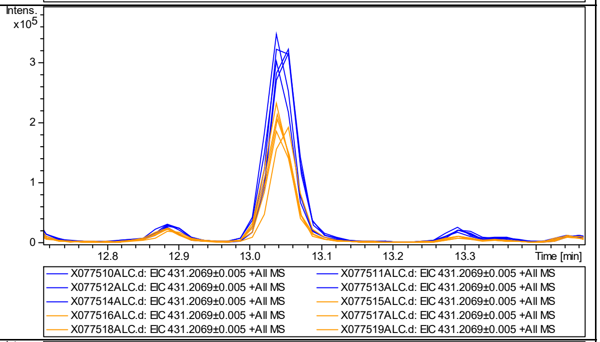
**AP12**	13.71	24	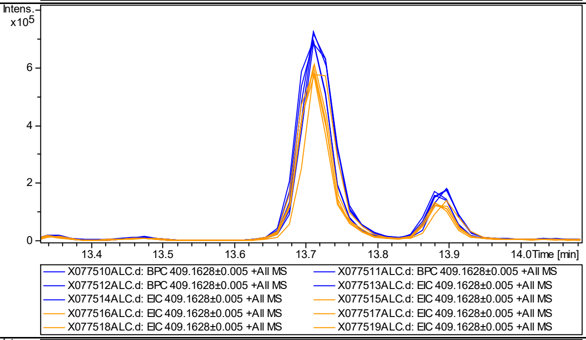
**AP13**	13.87	23	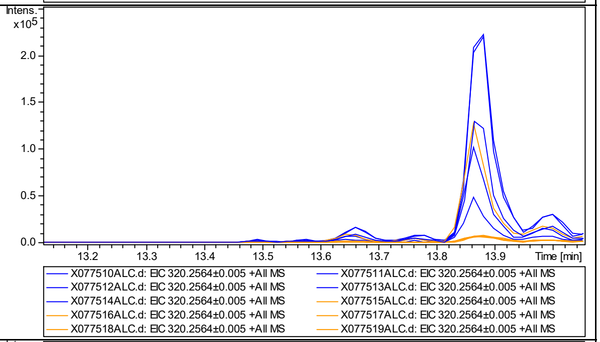
**AP14**	13.9	6	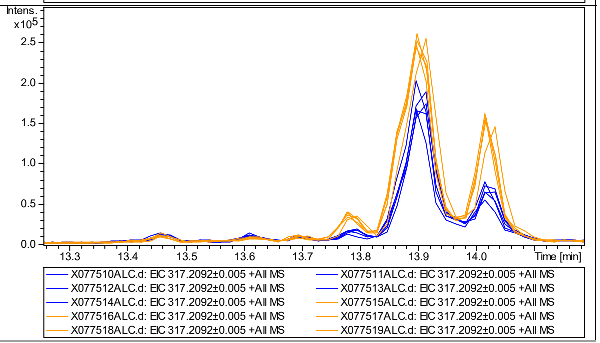

**AP15**	14.86	19	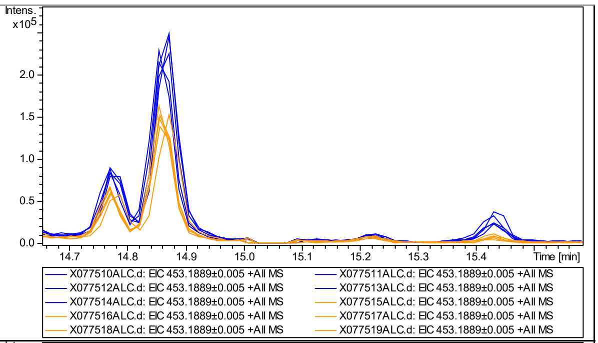
**AP16**	15.21	13	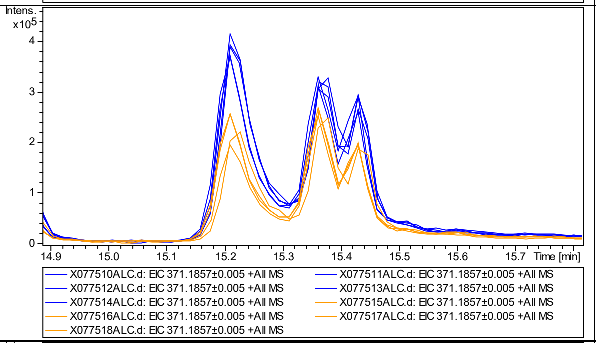
**AP17**	15.23	20	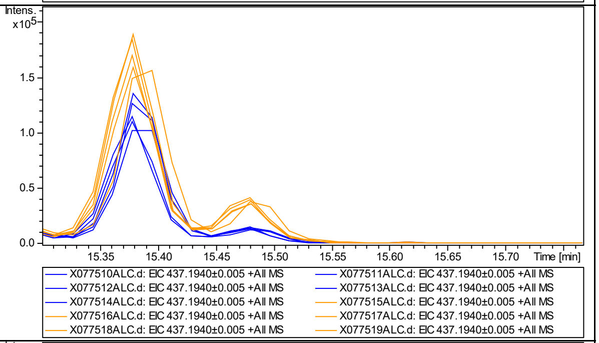
**AP18**	15.25<br>15.26	9	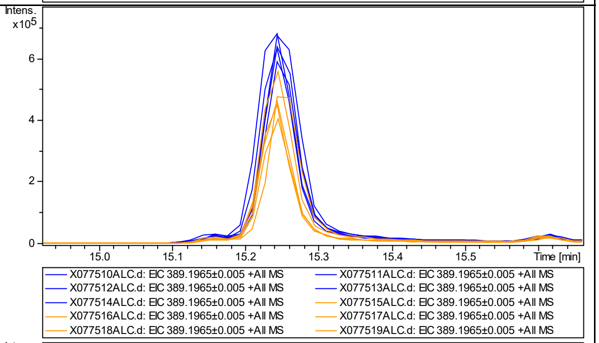
		25	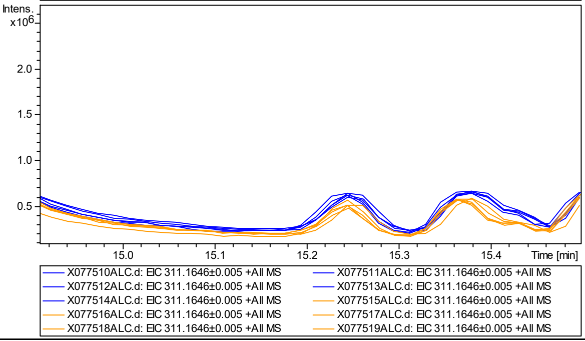

**AP19**	15.33	21	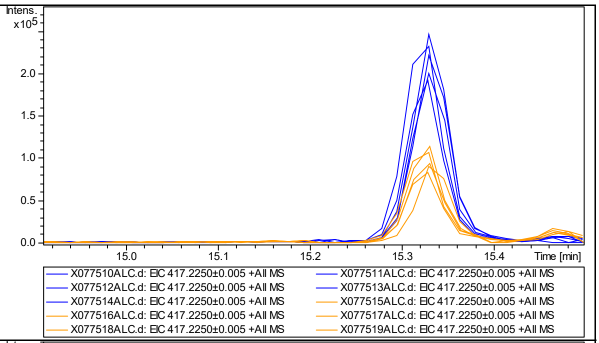
**AP20**	15.47	2	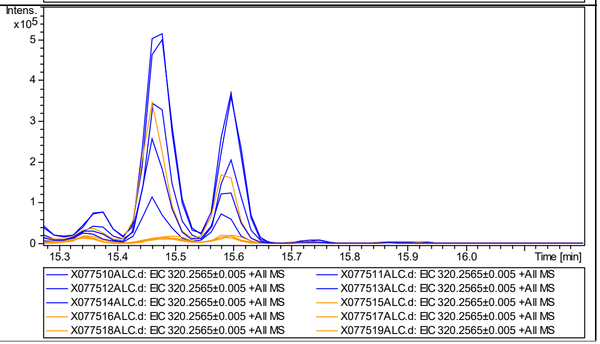
